# The novel distribution of intracellular and extracellular flavonoids produced by *Aspergillus* sp. Gbtc 2, an endophytic fungus from *Ginkgo biloba* root

**DOI:** 10.3389/fmicb.2022.972294

**Published:** 2022-10-26

**Authors:** Xinhong Wu, Kai Zou, Xueduan Liu, Shaodong Fu, Shuangfei Zhang, Zhenchun Duan, Jin Zhou, Yili Liang

**Affiliations:** ^1^School of Minerals Processing and Bioengineering, Central South University, Changsha, China; ^2^Key Laboratory of Biometallurgy, Ministry of Education, Changsha, Hunan, China; ^3^College of Advanced Materials Engineering, Jiaxing Nanhu University, Jiaxing, Zhejiang, China

**Keywords:** *Ginkgo biloba*, endophytic fungi, pathway, Liquid chromatography–tandem mass spectrometry, flavonoids

## Abstract

Here, we reported a *Ginkgo* endophyte, *Aspergillus* sp. Gbtc 2, isolated from the root tissue. Its flavonoid biosynthesis pathway was reconstructed, the effect of phenylalanine on the production of flavonoids was explored, and the flavonoid metabolites were identified with the high-resolution Liquid chromatography–mass spectrometry (LC–MS). Some essential genes were annotated to form the upstream of the complete biosynthesis pathway, indicating that *Aspergillus* sp. Gbtc 2 has the ability to synthesize the C6–C3–C6 flavonoid monomers. HPLC results showed that adding an appropriate amount of phenylalanine could promote the production of flavonoids by *Aspergillus* Gbtc 2. LC–MS results depicted a significant difference in many flavonoids between intracellularly and extracellularly. Most of the flavonoids gathered in the cell contained glycosylation groups, while almost all components with multiple hydroxyls showed much higher concentrations extracellularly than intracellularly; they likely have different biological functions. A variety of these substances can be mapped back to the pathway pattern of flavonoid biosynthesis and prove the ability of flavonoid production once again. This study expanded the information on flavonoid biosynthesis in *Aspergillus* and provided a solid theoretical basis for developing the fungi into genetically engineered strains undertaking flavonoid industrialized production.

## Introduction

As a necessary traditional medicine, *Ginkgo* flavonoids can be used to treat cardiovascular and cerebrovascular disease, Alzheimer’s disease, and other diseases (Eliana et al., 2013; Qian et al., 2016). However, the flavonoid content in *Ginkgo* extract is extremely low, which is challenging to meet the growing market demand (Isah, 2015). Due to the long-term co-evolution, plants and endophytic fungi may share some functional enzymes in the metabolic process (Teimoori-Boghsani et al., 2019; Wang H. et al., 2022). Endophytic fungi have been shown to indirectly or directly synthesize some intermediates of host plants (Jia et al., 2016; Ferreira et al., 2021), participating in plant signal pathways and assisting plants in completing physiological and biochemical processes (Shymanovich et al., 2015).

The taxol production provides a new direction for producing flavonoids by endophytic fungi (Cheng et al., 2018). In addition, endophytic fungi act as activators of plant host and synthesize active substances. A typical example is that *Aspergillus niger* could stimulate the rapid accumulation of taxol in the suspension cells of *Taxus* (Wang et al., 2001). In recent years, some studies on the metabolites of endophytic fungi in *Ginkgo biloba* have illustrated that *Penicillium* and *Mucor Circinelloides* SN2017 can produce flavonoids with anticancer and antioxidant activities (Trisuwan et al., 2010; Yuan et al., 2014; Ferreira et al., 2021). Moreover, in *Aspergillus niger* 13/5, substrate 7-hydroxyflavanone was transformed into 7-hydroxyflavone by dehydrogenase at C-2 and C-3 positions with a biotransformation rate up to 98% (Edyta and Tomasz, 2012). A variety of endophytic fungi also are isolated from *Ginkgo* and confirmed to have the ability to produce flavonoids (Qiu et al., 2010). It is reported that the flavonoid accumulation in suspension cells of *Ginkgo* can be induced by the abscisic acid (ABA) from fungal endophytes ((Hao et al., 2010)), which pushed us not to ignore the potential effect of endophytes in flavonoid synthesis and coevolution.

The flavonoid biosynthesis in plants mainly originates from the metabolic pathway of phenylalanine (Kazuki et al., 2013; Deng et al., 2021). Phenylalanine is catalyzed by phenylalanine ammonia-lyase (PLA) key enzymes to produce cinnamic acid, coumaroyl acid, and caffeoyl acid, and then, they are further catalyzed by 4-coumaric acid CoA ligase (4CL) enzymes to produce a variety of phenylalanine deamination derivatives, including cinnamoyl-CoA, p-coumaroyl-CoA, caffeoyl-CoA, and feruloyl-CoA. The upstream of this pathway mainly forms the basic C6-C3-C6 skeleton of flavonoids, which was transformed into a variety of chalcones by the chalcone synthase (CHS; Wang et al., 2020). Further, chalcones are condensed by chalcone isomerase (CHI) to form the parent structure of flavonoids. With flavone synthase (FNS), isoflavone synthase (IFS), flavonol synthase (FLS), and flavanone-3-hydroxylase (F3H), they are transformed into flavones, isoflavones, flavonols, and dihydroflavonols, respectively, (Xiaojia et al., 2017). In another branch of the pathway, dihydroflavonols are catalyzed by dihydroflavanol-4 reductase (DFR) to produce colorless anthocyanins, which are then catalyzed by anthocyanin synthase (ANS) and colorless anthocyanin reductase (LAR) to produce anthocyanins and flavones, respectively (Xie et al., 2003). Endophytic fungi and host plants symbiotically form a mutually beneficial relationship. In general, 4CL, CHS, CHI, and DFR were key functional enzymes for flavonoid biosynthesis in plants and endophytic fungi (Petit et al., 2007; Dao et al., 2011; Kaltenbach et al., 2018). At present, the biosynthetic pathway of flavonoids in plants has been clearly studied (Sajid et al., 2021). However, compared with higher host plants, endophytic fungi have the problem that some functional enzyme genes cannot be expressed. Therefore, the biosynthetic pathway of flavonoids in endophytic fungi was not necessarily the same as that in plants. The target product was still obtained by skipping the relevant enzymes in a certain pathway during flavonoid biosynthesis (Zang et al., 2019). In addition, the flavonoid biosynthesis pathway in plants becomes more complex due to the influence of various environmental factors, and this process may synthesize many by-products that are difficult to be controlled artificially (Pandey et al., 2016; Marsafari et al., 2020). Endophytic fungi are microorganisms, which have an advantage in reconstituting the biosynthetic pathway of flavonoids *in vitro* and controlling the reaction conditions accurately (Sajid et al., 2021). Moreover, microbial fermentation can form large-scale production in a short time, which is more suitable for industrial applications.

Extracting flavonoids directly from wild *Ginkgo* has the problems of long growth cycle, low extraction efficiency, high extraction cost, and non-renewable resources (Jiang et al., 2005). In this study, wild *Ginkgo* roots were sampled to isolate endophytes. Based on genomic analysis and metabolites identification, the flavonoid biosynthesis pathway of *Aspergillus* sp. Gbtc 2 was predicted and reconstructed. The intracellular and extracellular flavonoid products of *Aspergillus* sp. Gbtc 2 were detected and analyzed by Liquid Chromatography–Tandem Mass Spectrometry (LC–MS). At the same time, the effect of a certain amount of phenylalanine on the flavonoid synthesis of *Aspergillus* sp. Gbtc 2 was explored. Therefore, if *Ginkgo* flavonoids’ microbial source is reliable, it is expected to produce *Ginkgo* flavonoids through *Aspergillus* sp. Gbtc 2 fermentation. This method can significantly increase the yield of *Ginkgo* flavonoids and protect the resources of *Ginkgo*, so as to provide a theoretical basis for the subsequent industrialization of flavonoid synthesis by genetic engineering fungi.

## Materials and methods

### Endophytes isolation and genome extraction

The endophytic fungus was isolated from the root tissue of wild *Ginkgo biloba*, which grew in Linyi City, Shandong Province, China. The detailed location was 34°36′34” N, 118°12′8″ E with an altitude of 40 m. On October 13, 2019, the healthy wild *Ginkgo* tree with the largest DBH (0.64 m) in this area was chosen to collect the root materials. Evenly located around the tree, three locations were selected to collect the samples. Several root tissues of 2 cm diameter were cut off from the part of the tree, which was 1.5 m horizontally away for the trunk and 1 m deep underground. Then mixed to make a composite sample, which was used for endophyte isolation after strict surface disinfection. The specific surface disinfection methods were as follows: a. the rhizosphere soil and other attachments were completely removed by the brush and thoroughly washed with water; b. the samples were rinsed successively with 70% ethanol for 2 min, 5.25% sodium hypochlorite for 4 min, and sterile distilled water for 5 times; and c. the last eluent was used as a blank control for the verification experiment of thorough surface disinfection. Endophytic fungus was cultured on the Potato Dextrose Agar (PDA) medium at 25°C for 7–10 days. Hexadecyl Trimethyl Ammonium Bromide (CTAB) was chosen to extract the genomic DNA. Approximate 200 mg of fungal mycelia were homogenized and pestled with liquid nitrogen, successively followed by DNA extraction and purification as per the manufacturer’s recommendations. The concentration and purity were determined on a NanoDrop ND-1000 spectrophotometer (NanoDrop Technologies, Wilmington, United States). 1% (*w*/*v*) TAE-agarose gel stained with Elution Buffer (EB) was also used to detect DNA quality (Lei et al., 2021). A total of two DNA samples were extracted, one for BGISEQ-500 sequencing and the other for Pacbio Sequel sequencing. The 18S rRNA sequences of fungi and the similar sequences aligned with them in Genebank were analyzed by ClustalW (Juárez-Hernández et al., 2021). The method was neighbor-joining (NJ), the model was Kimura 2-parameter model, and the phylogenetic tree was constructed with MEGA X (Kumar et al., 2018). Bootstrap support was shown for cases in which the value was greater than 50% based on 1,000 replications.

### Library construction and sequencing

The pair-ended sequencing library was built as follows: (a) 1 μg DNA samples were fragmented in Covaris M220 focused ultrasonicator (Covaris, Inc.); (b) magnetic beads were used to screen DNA fragments with the size of 200 ~ 400 bp; (c) Qbit 2.0 was used to quantify the genomic libraries after screening and purification; (d) the DNA end was repaired with adding base A at the 3′ end; (e) the adapter sequence was stably connected to the DNA at a specific temperature; (f) DNA was further purified by magnetic beads, and then dissolved in EB; and (g) the fragment size of the library was confirmed by Agilent 2,100 Bioanalyzer instrument. To obtain more accurate genomes, another 20 μg DNA extracts were subjected to DNA interruption, terminal repair, linker sequence connection, fragment purification, primer hybridization, and polymerase binding, and finally determined as the library for single-molecule sequencing. Pair-ended sequencing was completed on the BGISEQ-500 platform (BGI, Shenzhen, China), and single-molecule sequencing was completed on the Pacbio Sequel platform.

### Genome assembly and functional annotation

Clean data were obtained by removing the adapter, ploy-N, and low-quality reads from raw data. The genomic assembly was accomplished in Canu (v1.9) using the single-molecule sequencing data (Koren et al., 2017), and then corrected with the pair-ended clean reads in NextPolish (v1.0.5; Hu et al., 2020). After that, BUSCO (V4.1.2) was employed to evaluate the assembly quality (Simão et al., 2015). GeneMark-ES (v4.48_3.60_lic) was run to predict the gene composition (Sayers et al., 2022), and the obtained CDS sequences were functionally annotated according to the universal database resources. The diamond (v2.0.4) program was used to do blasting against the NR database (Buchfink et al., 2015). KAAS system was used for annotation and pathway mapping (Moriya et al., 2007), with the default e-value 1E-6. The COG classification of all predicted amino acid sequences was analyzed in the eggnog-mapper (Huerta-Cepas et al., 2017, 2019). Subsequently, the metabolic pathway was reconstructed in iPath3 (Darzi et al., 2018).

### Liquid chromatography–tandem mass spectrometry sample preparation

The LC–MS samples were prepared by Potato Dextrose Broth (PDB) liquid culture method. The mycelium and spores were scraped from the surface of the medium by a sterile blade and suspended in a centrifuge tube. 10 μl suspension (~ 10^6^ cells/ml) was inoculated into 500 ml sterile PDB. After inoculation, all liquid media were placed in a shaking incubator at 140 rpm at 25°C for 14 days. Four biological replicates were set up in this experiment, one of which was used to prepare QC samples. The blank control group was set synchronously to filter the bottom noise during LC–MS detection. Three biological repetitions were filtered with sterile gauze and the mycelium was separated from the culture medium. The mycelium and culture medium were put into BLK-FD-0.5 Vacuum Freeze Dryer (Jiangsu Bolike Refrigeration Technology, Changzhou, Jiangsu) for freeze-drying. The freeze-dried samples were ground at −4°C for 15 min by a grinding instrument with a power set to 30 Hz. Then, the sample was collected in a centrifuge tube and added 100 ml of 70% methanol. The QC sample was a mixture of mycelium and culture medium and followed the same experimental treatment. The blank control was directly freeze-dried from the blank medium, and the follow-up operation was the same as the three biological replicates. All the samples added with methanol were extracted overnight at 4°C, during which they were whirled three times. 1.0 ml of mycelium extract (intracellular metabolites), culture medium extract (extracellular metabolites), blank sample extract, and QC extract were put into the centrifuge tube. The sample was centrifuged for 15 min at 10000 × *g* within 4°C temperature control. The supernatant was filtered through a 0.22 μm membrane before LC–MS analysis. In this experiment, substances in mycelium were defined as intracellular flavonoid metabolites, while substances secreted into culture medium were defined as extracellular flavonoid metabolites.

### Liquid chromatography–tandem mass spectrometry conditions

Chromatographic separation was carried out on Shim-pack UFLC SHIMADZU CBM30A liquid chromatographic system (Shimadzu, Duisburg, Germany) equipped with a Water ACQUITY UPLC HSS T3 C18 column (1.8 μm, 2.1 mm * 100 mm) at 35°C. A flow rate of 0.4 ml/min was chosen to use while 0.04% acetic water (A) and Acetonitrile (B) with 0.04% acetic acid comprised the mobile phase. Gradient elution program was optimized as follows: 0 ~ 11.0 min, 5% → 95% B; 11.0 ~ 12.0 min, 95% → 95% B; 12.0 ~ 12.1 min, 95% → 5% B; 12.1 ~ 15.0 min, and 5% → 5% B. The injection volume was 5 μl. MS was carried out on an Applied Biosystems 4,500 QTRAP system (AB SCIEX Technologies, United States), equipped with electrospray ionization (ESI) and UHPLC system to scan 100 to 1,500 molecular weight parent ions at 550°C. Other MS parameters were set as follows: MS voltage: 5,500 V; curtain gas (CUR): 25 psi; collision-activated dissociation (CAD): high; declustering potential (DP) and collision energy (CE): specific optimization (Chen et al., 2013).

### Liquid chromatography–tandem mass spectrometry quality control and flavonoid identification

The raw data output from LC–MS was pretreated by Analyst 1.6.1 (AB SCIEX Technologies, United States; Chen et al., 2009), including peak recognition, alignment, calibration of the internal standard, filtering, and normalization to total area. Analyst 1.6.1 (AB SCIEX Technologies, United States) was also used to visualize the target components in a two-stage mass-to-charge ratio map as recommended. The repeatability of metabolite extraction and instrument detection can be judged by analyzing the overlap of mass spectrometry total ion chromatograms (TIC diagram). Based on the local MWDB database (Wuhan Metware Biotechnology Co., Ltd., Wuhan, Hubei) and metabolic public databases, like MassBank (Horai et al., 2010), KNAPSAcK (Sur et al., 2022), HMDB (Wishart et al., 2018), METLIN (Domingo-Almenara et al., 2018), and so on, the components were identified based on fragment ion information.

### HPLC sample preparation

Hundred ml sterile PDB liquid medium was set as the blank control group. The samples with 0.0 mg/ml, 1.0 g/l, 2.0 g/l, 5.0 g/l, and 10.0 g/l exogenous phenylalanine were set as the experimental group. Three replicates were set in the blank control and experimental groups. In this experiment, the total flavonoids in the whole fermentation broth were determined, and the mycelium and culture medium were not separated. The methods of inoculation, culture, freeze-drying, and extraction were the same as described in “Liquid chromatography–tandem mass spectrometry sample preparation”.

Accurately weigh 10 mg quercetin, 20 mg kaempferol, and 10 mg isorhamnetin, and three standards were mixed. The standard stock solution was obtained by using 25 ml of methanol at constant volume. The standard stock solution was diluted to 2 ×, 5 ×, 10 ×, 25 ×, and 50 × to prepare the standard curve. After determination by HPLC, the peak areas (X) of quercetin, kaempferol, and isorhamnetin were substituted into the standard curve formula to obtain the corresponding flavone concentrations, which were expressed in Q, K, and I respectively, so as to calculate the flavone content of each sample. The formula was as follows: C = 2.51Q + 2.64 K + 2.39I.

The supernatant was filtered through a 0.22 μm membrane before HPLC analysis. Analysis was carried out using an LC-20AT liquid chromatographic system (Shimadzu, Japan). HPLC separations were accomplished on a 5.0 um, 150 mm * 6.0 mm Shimadzu ODS C18 column (Shimadzu, Japan) at 35°C. A flow rate of 1.0 ml/min was chosen to use while 0.04% acetic water (A) and Acetonitrile (B) with 0.04% acetic acid comprised the mobile phase. The optimized gradient elution program was the same as described in “Liquid chromatography–tandem mass spectrometry sample preparation”. The injection volume was 10 μl. A wavelength of 360 nm was selected for quantification.

## Results

### Strain identification

**Figure 1:**
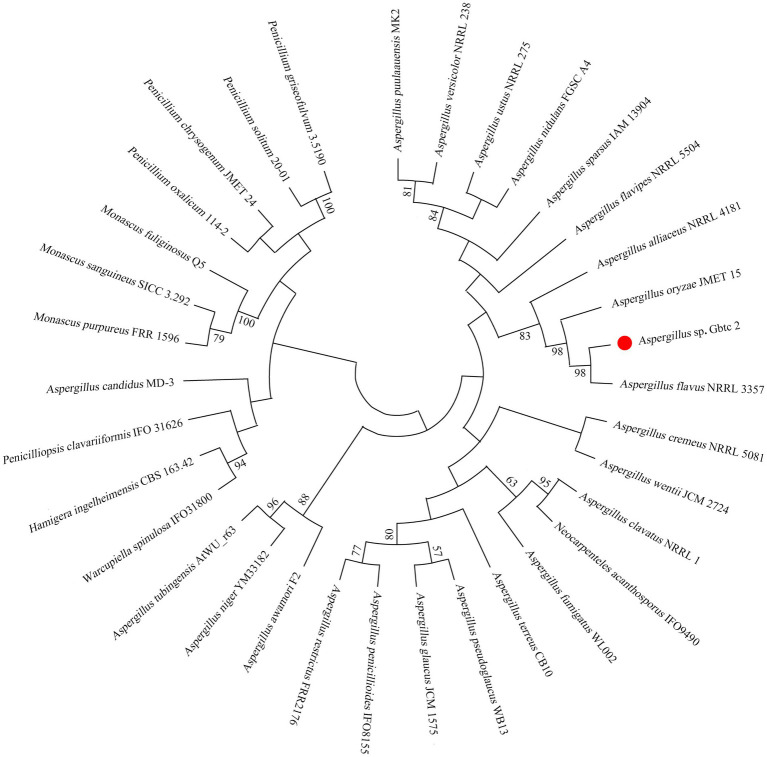
Molecular phylogenetic analysis of fungal isolates. The numbers at the nodes indicate the levels of bootstrap support based on Kimura distance and the neighbor-joining method. Bootstrap support was shown for cases in which the value was greater than 50% based on 1,000 replications.

After the last elution solution was coated on the culture medium, the growth of sterile colony showed that the surface was completely disinfected. The endophytic fungus was isolated from the root of *Ginkgo* and named *Aspergillus* sp. Gbtc 2. According to the BLASTN result of the 18S rRNA region, Gbtc 2 is the most similar to *Aspergillus flavus* (98%). The phylogenetic tree was built in Figure 1 based on 34 18S rRNA sequences (the accession numbers of 33 reference sequences were shown in Supplementary Table S1) by the neighbor-joining (NJ) method in MEGA X. Combined with phylogenetic analysis and sequence homology, the strain Gbtc 2 was identified as *Aspergillus flavus*. However, through KEGG and NR database annotation, no gene related to aflatoxins synthesis was found in *Aspergillus* sp. Gbtc 2.

### Sequencing, assembly, and functional annotation

As the detailed information listed in Table 1, a total of 34,022,048 clean reads (BGISEQ-500, PE150) were obtained to include 5,097,121,946 clean bases for Gbtc 2, and its Q20 was 96.58%. Considering that fungi generally have a larger genome than bacteria, the introduction of the Single Molecule, Real-Time (SMRT) Sequencing technology was necessary to improve the assembly quality. Here, next polished with the pair-ended PE150 reads, the Pacbio Sequel output was assembled into the high-quality draft genome of Gbtc 2 (16 contigs). Gbtc 2 had a genome of 37,929,866 bp with an N50 of 4,075,796 bp. The G + C content was 47.45%, similar to the strains in the GeneBank database. On the other hand, the protein sequences of the model strain homologous to Gbtc 2 were selected for BUSCO evaluation, and the accession numbers were XP_043133698.1 (*Aspergillus chevalieri*), XP_041140511.1 (*Aspergillus flavus* NRRL3357), XP_748963.1 (*Aspergillus fumigatus* Af293), XP_041544984.1 (*Aspergillus luchuensis*), XP_001821344.1 (*Aspergillus oryzae* RIB40), and XP_041556067.1 (*Aspergillus puulaauensis*), respectively. The BUSCO held 74.5% integrity assessment, indicating that the genome assembly results were acceptable (Supplementary Figure S1).

**Table 1:** The statistics of gene assembly and function annotation.

Attributes	Characteristic
Clean reads	34,022,048
Clean base	5,097,121,946
Q20(%)	96.58
Scaffolds	16
N50	4,075,796
Total base	37,929,866
GC content (%)	47.45
CDS	13,307
rRNA genes	68
tRNA genes	290
Genes assigned to KEGG	3,902
Genes assigned to NR	13,216
Genes assigned to COG/KOG	10,739
All^1^	3,899
At least one^2^	13,216
Percentage^3^ (%)	99.32

A total of 13,307 CDSs, as well as 68 rRNA and 290 tRNA genes, were predicted in the genome. Of these, 3,902 CDSs were successfully annotated in KEGG database, while 10,739 sequences in COG/KOG database and 13,216 sequences in NR database. Commonly, the NR database depicted the highest annotation rate. The global annotation rate of this fungus reached 99.32% (Table 1).

The datasets presented in this study can be viewed in online repositories. The names of the repository/repositories and accession number can be found at: https://www.ncbi.nlm.nih.gov/bioproject/PRJNA870667.

### Reconstruction of Gbtc 2 flavonoid metabolic pathway

**Figure 2:**
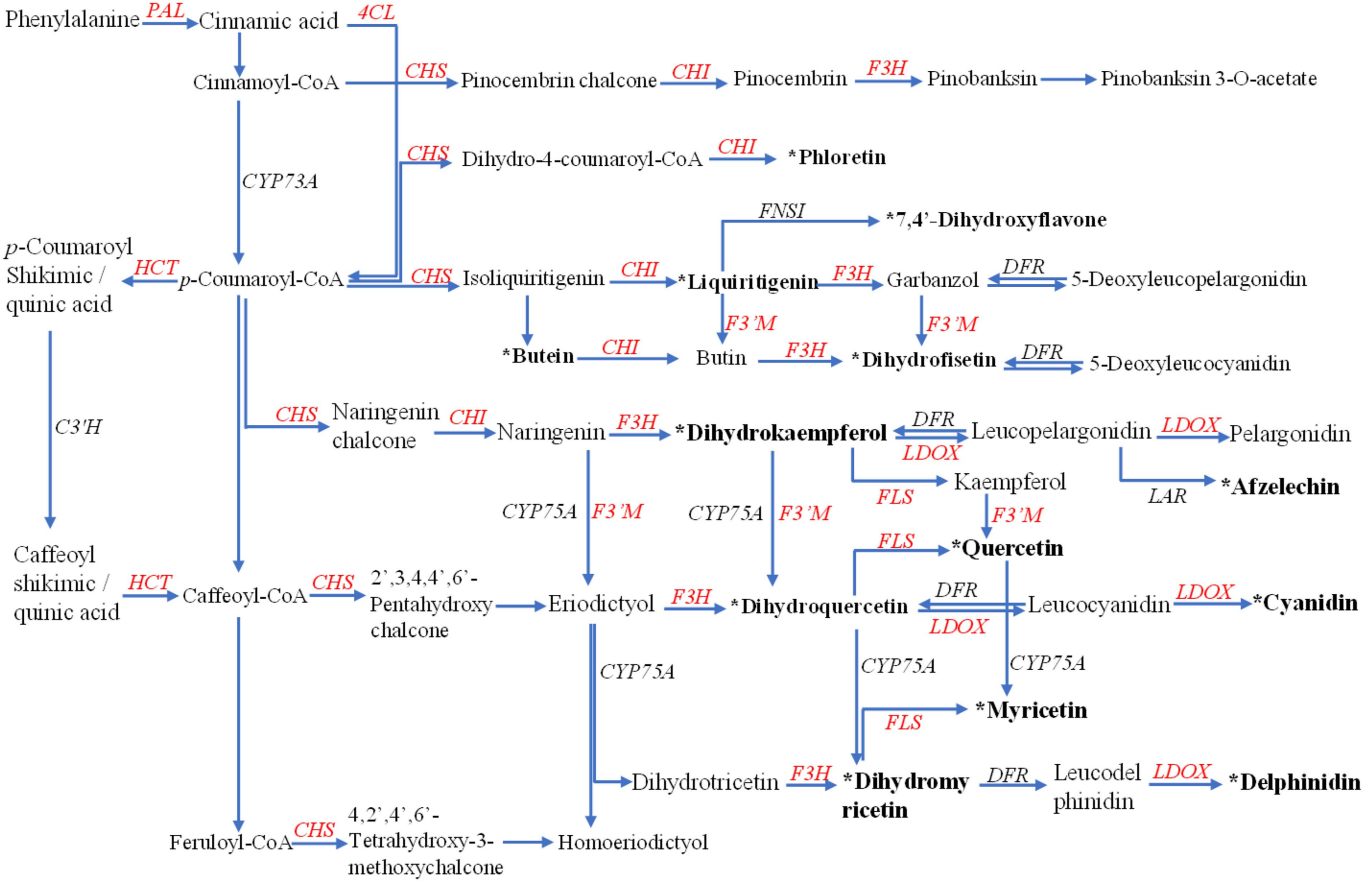
The remodeling of the flavonoid metabolism pathway in *Aspergillus* sp. Gbtc 2. The enzymes annotated in Gbtc 2’s genome are marked in red, including phenylalanine ammonia-lyase (PAL), shikimate O-hydroxycinnamoyltransferase (HTC), 4-coumarate: CoA ligase (4CL), chalcone synthase (CHS), chalcone isomerase (CHI), flavanone 3-hydroxylase (F3H), flavonoid 3′-monooxygenase (F3′M), flavonol synthase (FLS), and leucoanthocyanidin dioxygenase (LDOX); while the unannotated in black, including trans-cinnamate 4-monooxygenase (CYP73A), 5-O-(4-coumaroyl)-D-quinate 3′-monooxygenase (C3′H), flavonoid 3′,5′-hydroxylase (CYP75A), flavone synthase I (FNS I), dihydroflavonol 4-reductase (DFR), and leucoanthocyanidin reductase (LAR). The flavonoids identified in the metabolites of Gbtc 2 are shown in bold black text with an asterisk.

The anabolism of flavonoids generally exists in most plants. It was the primary way for plants to resist excessive ultraviolet radiation and oxidative damage (Sun et al., 2020). At the same time, flavonoids also have a particular ability to resist diseases and pests (Erb and Kliebenstein, 2020; Singh et al., 2021). Here, we reconstructed the flavonoid biosynthetic pathway of Gbtc 2 according to the model of flavonoids in plants, although it may not be suitable for endophytic fungi. As shown in Figure 2, Gbtc 2 has almost the complete upstream of flavonoid synthesis to form the basic C6–C3–C6 flavonoid monomer. In the KEGG database, the coding genes corresponding to PAL, 4CL, and CHS were annotated. Under the catalysis of CHS, cinnamoyl-CoA, p-coumaroyl-CoA, caffeoyl-CoA, and feruloyl-CoA were converted to pinocembrin chalcone, naringenin chalcone, eriodictyol chalcone, and homoeriodictyol chalcone, respectively. Since no gene related to TAL was annotated in the sequencing results, it is indicated that Gbtc 2 may have the ability to synthesize chalcone from phenylalanine. Secondly, when comparing the sequences of unknown functions in the NR database, multiple downstream functional enzyme genes were found to be related to the synthesis and derivation of flavonoids, such as *CHI*, *F3H*, colorless anthocyanin oxygenase gene (*LDOX*), and flavonoid-3′-monooxygenase gene (*F3′M*) (Enzyme genes with gene_ID involved in flavonoid metabolism pathway in *Aspergillus* sp. Gbtc 2 were listed in Supplementary Table S2). Of them, CHI was responsible for converting various chalcone into flavonoid monomers, such as pinocembrin, liquiritigenin, butin, and naringenin, while F3H catalyzes these flavonoid monomers into dihydroflavonols. Notably, the genes encoding flavonoid 3′-monooxygenase (F3′M) have been successfully annotated in the assembly (Figure 2), which will lead to frequent synergistic transformations between flavonoid compounds, such as from kaempferol to quercetin. In conclusion, Gbtc 2 can be considered to have the potential to synthesize flavonoids independently, which points out the direction for metabolic detection and identification.

### Analysis of flavonoid metabolites

After filtering the blank control signal, there were relatively many kinds of metabolites in the sample. The positive ion and negative ion modes reflected the reliability of sample preparation and LC–MS detection (Supplementary Figure S2A). In the multi-peak diagram of MRM metabolite detection in multi-reaction monitoring mode, the samples seen in positive ion and negative ion modes are displayed, and the peaks of different colors represent different metabolites (Supplementary Figure S2B). A total of 71 flavonoids were identified based on the local and public databases, including 13 flavones, 4 isoflavones, 18 flavonols, 8 flavanones, 14 C-glycosyl flavonoids, 12 anthocyanins, and 2 procyanidins. Table 2 shows the top 30 substances in the signal intensity of flavonoids in Gbtc 2 mycelium and culture medium under the same conditions. The signal intensity of other flavonoids is listed in Supplementary Table S3. According to the descending order of total signal intensity, cyanin, afzelechin, dihydromyricetin, kaempferol 3-O-glucoside, di-O-methylquercetin, genistein, trifolin, and 4′-hydroxy-5,7-dimethoxyflavanone had higher total signal intensity. While components with weaker signal strength may be affected by detection conditions, there is no doubt that elements with higher signal strength really exist.

**Table 2:** The qualitative and relative quantitative table of flavonoids.

Rt (min)	Mr (Da)	Ionization mode	Compounds	Class	Culture medium	Mycelium
2.26	611	Protonated	Cyanin	F	1.51E+06	2.26E+05
3.29	274.084	[M–H]–	Afzelechin	D	1.14E+06	1.74E+05
3.36	320.053	[M–H]–	Dihydromyricetin	B	4.34E+05	1.28E+05
4.03	448.101	[M–H]–	Kaempferol 3-O-glucoside	B	9.00E+00	5.22E+05
5.76	330.1	[M–H]–	Di-O-methylquercetin	B	5.03E+04	4.45E+05
5.45	270.053	[M + H]+	Genistein	C	4.47E+05	9.00E+00
3.86	448.101	[M–H]–	Trifolin	B	9.00E+00	4.44E+05
6.58	300.1	[M–H]–	4′-Hydroxy-5,7-dimethoxyflavanone	D	6.68E+04	3.63E+05
3.05	474.1	[M–H]–	Pelargonidin O-acetylhexoside	F	1.92E+05	6.32E+04
3.7	448.101	[M–H]–	Cynaroside	A	9.00E+00	2.47E+05
4.83	286.048	[M + H]+	Orobol	C	2.11E+05	9.00E+00
3.05	948.1	[M + H]+	C-hexosyl-luteolin O-feruloyl-hexosyl-hexoside	E	1.36E+05	4.98E+04
2.94	756.1	[M + H]+	6-C-hexosyl-apigenin O-hexosyl-O-hexoside	E	1.15E+05	3.07E+04
4.43	288.063	[M–H]–	Dihydrokaempferol	B	9.47E+04	1.88E+03
4.87	256.074	[M–H]–	Liquiritigenin	D	9.00E+00	9.50E+04
2.87	548.1	[M–H]–	Peonidin O-malonylhexoside	F	4.12E+04	4.48E+04
6.84	402.132	[M + H]+	Nobiletin	A	2.13E+04	6.42E+04
3.93	608.4	[M + H]+	Chrysoeriol 7-O-rutinoside	A	9.00E+00	8.28E+04
3.99	610.19	[M–H]–	Hesperidin	D	4.54E+03	7.82E+04
3.61	432.1056	[M–H]–	Isovitexin	E	9.00E+00	8.11E+04
2.9	303.24	Protonated	Delphinidin	F	4.69E+04	3.16E+04
2.45	449.1	Protonated	Cyanidin 3-O-glucoside	F	3.60E+04	2.74E+04
7.32	372.121	[M + H]+	Tangeretin	A	1.75E+04	4.58E+04
4.09	610.19	[M + H]+	Neohesperidin	D	1.46E+04	4.81E+04
3.7	594.159	[M–H]–	Biorobin	B	6.63E+03	5.28E+04
4.39	318.038	[M + H]+	Myricetin	B	4.83E+04	1.18E+03
5.34	274.084	[M–H]–	Phloretin	D	9.00E+00	4.70E+04
4.37	270.053	[M–H]–	2′-Hydroxydaidzein	C	3.11E+04	1.18E+04
5.8	270.053	[M–H]–	Baicalein	A	4.21E+04	9.00E+00
3.51	610.153	[M + H]+	Rutin	B	3.37E+03	3.77E+04

A. Flavone; B. Flavonol; C. Isoflavone; D. Flavanones; E. Flavone C-glycosides; F. Anthocyanins.

The identification results of metabolites were added to the flavonoid metabolic pathway in “Reconstruction of Gbtc 2 flavonoid metabolic pathway” for joint mapping. As shown in Figures 2, a total of 13 metabolites were mapped into the existing flavonoid synthesis pathway, including dihydromyricetin, dihydrokaempferol, taxifolin, quercetin, myricetin, and afzelechin, etc. These metabolites play essential roles in flavonoid synthesis, proving that the existing plant-based flavonoid biosynthesis model may also be applicable to Gbtc 2.

**Figure 3:**
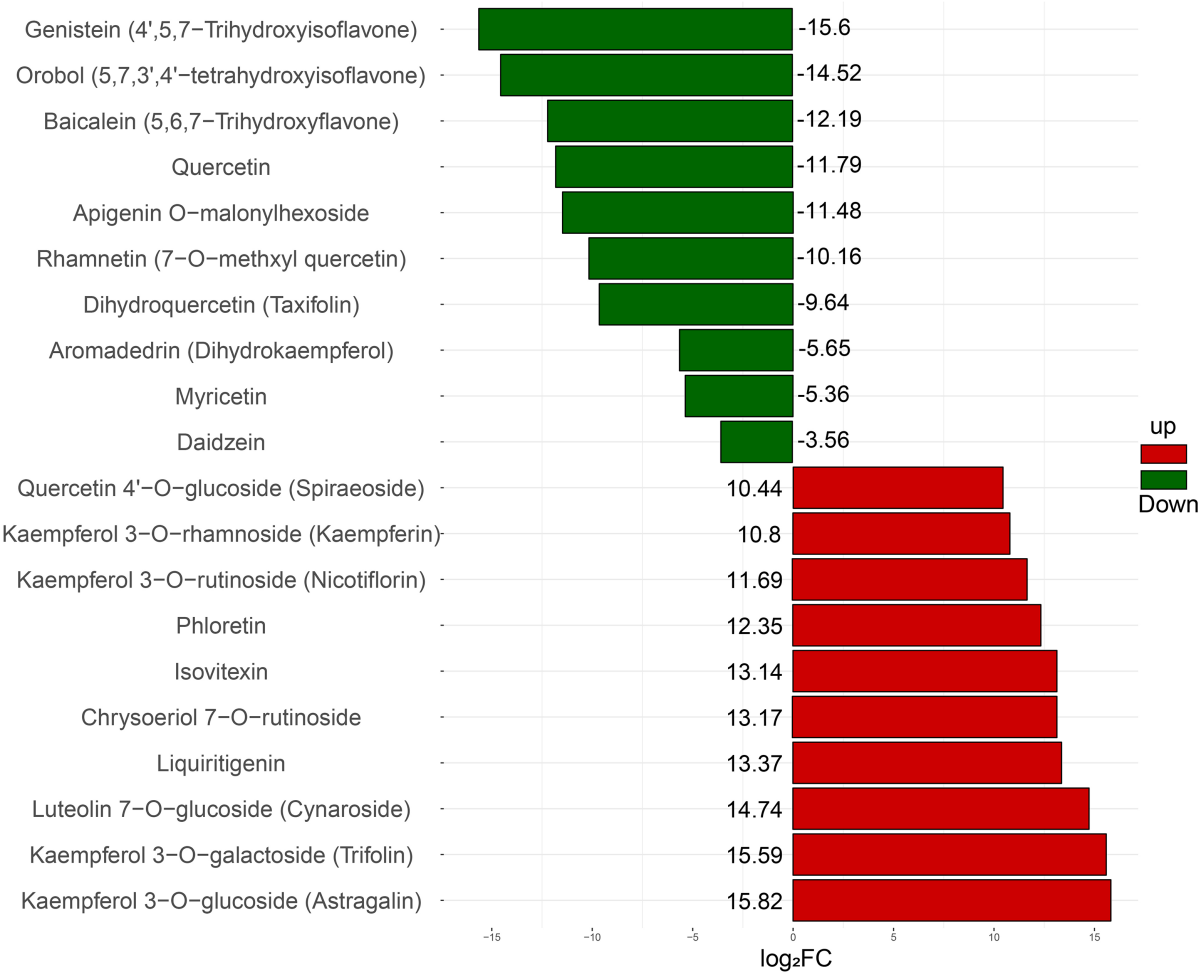
The relative concentration difference of intracellular and extracellular flavonoid metabolites. FC represents the ratio of the ion signal intensity of intracellular flavonoid metabolites to the ion signal intensity of extracellular flavonoid metabolites. The up-regulated metabolites were relatively large amounts of intracellular aggregation, while the downregulated metabolites were relatively large amounts of extracellular aggregation.

In addition, the relative concentrations of different flavonoids were quite different in intracellular and extracellular. Based on the logarithm of the ratio (log_2_FC) of metabolites’ intracellular and extracellular ion signal intensity, 20 metabolites with the highest difference in intracellular and extracellular concentrations are shown in Figure 3. The results showed that genistein, orobol, baicalein, quercetin, apigenin O-malonylhexoside, rhamnetin, taxifolin, and other metabolites were much higher extracellularly than intracellularly. Meanwhile, astragalin, trifolin, cynaroside, liquiritigenin, chrysoeriol-7-O-rutinoside, isovitexin, phloretin, kaempferol-3-O-rhamnoside, kaempferin, spiraeoside, and other metabolites mainly accumulated intracellularly. Moreover, most of the flavonoids gathered intracellular contain glycosylation groups, such as astragalin, trifolin, cynaroside, kaempferol, etc., while almost all metabolites with much higher concentration extracellular than intracellular were flavonoid monomers with multiple hydroxyls.

### Effects of phenylalanine on total flavonoids

According to the relationship between peak area (X) and concentration (Q, K, I) of quercetin, kaempferol, and isorhamnetin, the standard curve was Q = 2.5072 × 10^−7^ × X + 2.9841 × 10^−4^ (R^2^ = 0.9981), K = 1.9469 × 10^−7^ × X + 3.1478 × 10^−4^ (R^2^ = 0.9987) and I = 1.2007 × 10^−7^ × X + 3.2847 × 10^−4^ (*R*^2^ = 0.9989). The concentrations of quercetin, kaempferol, and isorhamnetin in samples were determined by HPLC to calculate the concentration of total flavonoids in each sample (Supplementary Figure S3). Since isorhamnetin was not detected in all samples, it was not counted in Supplementary Table S4.

**Figure 4:**
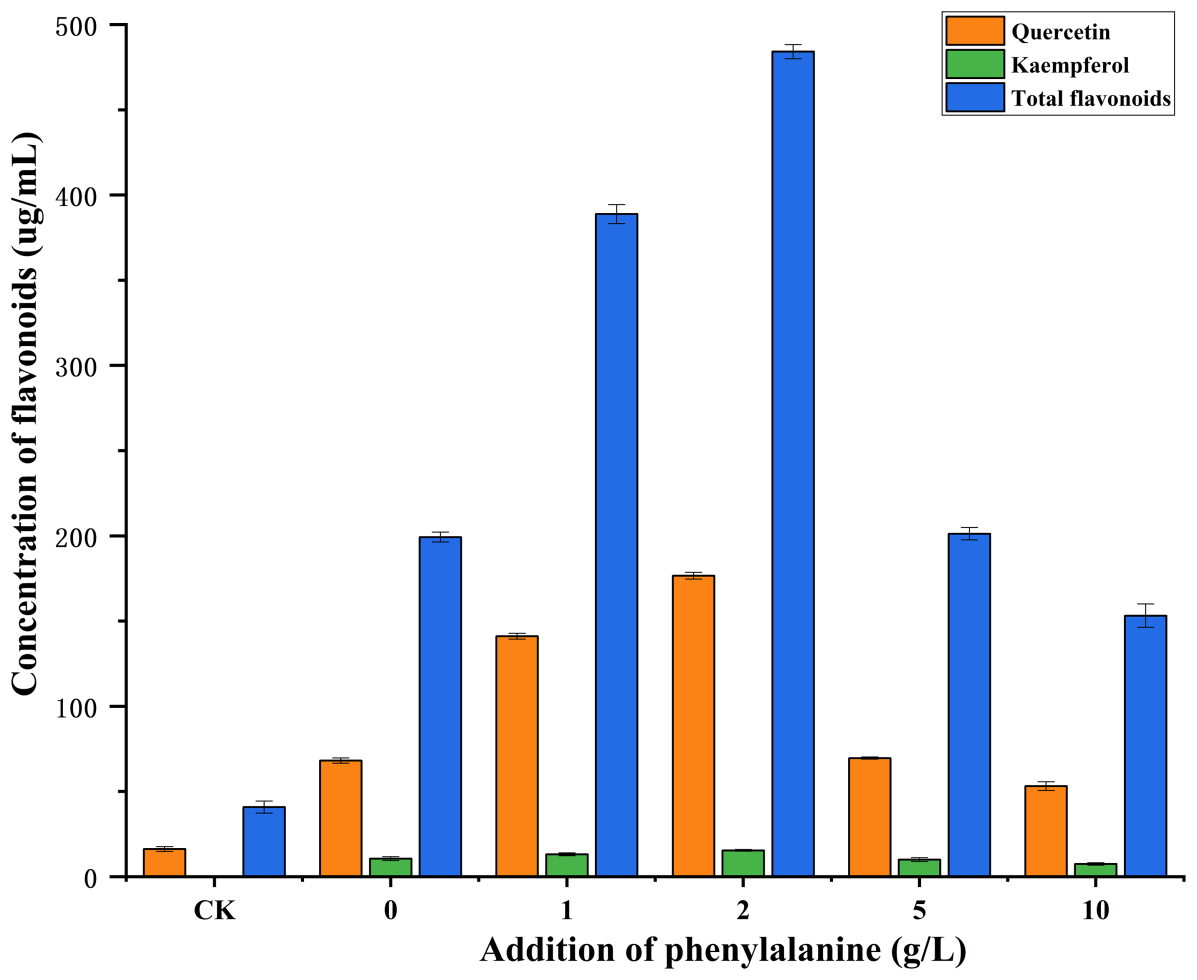
Comparison of flavonoid content between phenylalanine added/non-phenylalanine added treatment and PDB blank control group. CK stands for PDB blank control group; 0, 1, 2, 5, and 10 represent the amount of phenylalanine.

PDB medium was widely used to culture fungi. In this experiment, Gbtc 2 was screened out on PDA medium, and Gbtc 2 was verified to grow well on the PDB medium. Since the main component of PDB medium contains higher plant potato, the flavonoid content compounds in the blank PDB medium were tested to compare the flavonoid content in the fermentation broth cultured with Gbtc 2. It was verified that Gbtc 2 indeed has the ability to produce flavonoids. However, we did not choose *Ginkgo biloba* as the experimental materials, because *Ginkgo biloba* tissue also contains a large number of other active substances, and its components are more complex than PDB medium. In addition, *Ginkgo biloba* tissue also contains some toxic substances, such as ginkgolic acid, which are very likely to have adverse effects on the growth of Gbtc 2. In PDB blank medium (CK), quercetin accounted for the vast majority of total flavonoids, while kaempferol and isorhamnetin were almost absent (Supplementary Figure S4), and the contents of quercetin, kaempferol, and total flavonoids were 16.2672, 0.0121, and 40.8627 μg/ml, respectively. However, after inoculation with Gbtc 2, the contents of quercetin, kaempferol, and total flavonoids were significantly increased (Supplementary Figure S5), and the contents of quercetin, kaempferol, and total flavonoids were 68.1733, 10.6600, and 199.2575 μg/ml, respectively. At the same time, the addition of phenylalanine in PDB was 1.0 and 2.0 g/l, which greatly promoted the production of quercetin, thus increasing the yield of flavonoids. When the amount of phenylalanine was 2.0 g/l, the contents of quercetin, kaempferol, and total flavonoids reached the highest, 176.6467, 15.4267, and 484.1095 μg/ml, respectively. It shows that Gbtc 2 can make good use of phenylalanine to accumulate a large amount of coumaric acid, so as to metabolize and produce more flavonoids. However, when the amount of exogenous phenylalanine was 5.0 and 10.0 g/l, the content of total flavonoids in the fermentation broth was not significantly different from that of the total flavonoids without exogenous phenylalanine, and even its content was lower than that of the control group without phenylalanine. The experimental results show that adding a certain concentration of phenylalanine can promote the flavonoid yield of Gbtc 2, but adding excessive phenylalanine may inhibit the production of flavonoids by Gbtc 2 (Figure 4).

## Discussion

### Gbtc 2 and *ginkgo* root may share the secondary metabolic pathways of flavonoids

For a long time, the symbiosis and evolution of endophytic fungi and plant hosts have established a special relationship closely related to the production of active metabolites in plants (Toghueo, 2020). The *Ginkgo* host has a complete MEP pathway to synthesize IPP and DMAPP, while the IDI gene was only identified in the multiple isolates, indicating that these endophytic fungi may play a key role in regulating the mutual transformation of these two components (Liu et al., 2022). If the MEP pathway of *Ginkgo* comes from an endosymbiotic prokaryote as widely accepted, the MVK gene involved in the original MVA pathway of *Ginkgo* may have a non-highly-homologous replaceable copy, or it may be compensated by endophytes due to a long-term symbiotic relationship, like *Cellulomonas* sp. Gbtc_1 (Zou et al., 2021). Endophytic fungi were the production source of many known or unknown new bioactive metabolites (Queiroz and Santana, 2020; Aftab et al., 2021; Bezerra et al., 2021). In the study of *Aspergillus oryzae*, a complete phenylalanine flavonoid metabolic pathway with *CHS*-like genes as the core was found, and the functional enzymes in the pathway were also verified (Juvvadi et al., 2005). Moreover, information exchanged between *Ginkgo* and endophytes may be carried out through LTR-RT genetic elements. *Streptomyces* sp. Gbtc 1 was also shown to contain genes for key functional enzymes in synthesis pathway of natural products and local secondary metabolites similar to *Ginkgo* host (Zou et al., 2021). *Mucor circinelloides* DF20 has been reported to promote biosynthesis and accumulation of tanshinone in *Salvia miltiorrhiza* (Chen et al., 2021). The ability of Gbtc 2 to promote host flavonoid synthesis was inferred after comparing the Gbtc 2 genome to the published *Ginkgo* genome. Furthermore, potential functional complementarity, substitution, and genetic evolution exist between Gbtc 2 and host *Ginkgo*.

So far, few bacteria have been observed to possess PAL, 4CL, and CHS, which reflects that it was difficult for bacteria to produce chalcone directly (Moore et al., 2002). Gbtc 2 has the potential to produce chalcones because it carries these three genes at the same time. *CHI* homologous genes commonly exist in some fungi, myxomycetes, and γ-proteus. But microbes with *CHI* genes generally lack the upstream *CHS* homolog (Gensheimer and Mushegian, 2004), and our results supplement the lack of information in this regard. Endophytic fungi can produce new flavonols or metabolize glycosyl flavonoids into glycosyl flavonoid aglycones (Yan et al., 2013), and further participate in the flavonoid metabolism of plant hosts (Yuan et al., 2014). In this study, Gbtc 2 may derive downstream flavonoids by adding hydroxyl groups. Considering that most flavonoids, lignin, lignans, and hydroxycinnamic acid are synthesized from the same substrate (Zou et al., 2019), Gbtc 2 and *Ginkgo* host may be involved in the regulation of substance flow at the same time.

### Comparison of intracellular and extracellular differences in flavonoid production of Gbtc 2

Endophytic fungi are closely related to the ability of plants to produce flavonoids. Most of the functional enzymes related to flavonoid synthesis exist in fungi, including PAL, CHS, CHI, F3H, DFR, ANS, LAR, etc. (Xie et al., 2003; Li et al., 2012; Cheng et al., 2018). Traces of *Aspergillus oryzae* phenylpropane-flavonoid metabolic pathway indicated the ability to synthesize flavonoid substances (Juvvadi et al., 2005; Mohanta, 2020). Combined with the second-generation and third-generation sequencing techniques, the relatively complete genome of Gbtc 2 was constructed, and the entire flavonoid synthesis pathway was predicted. HPLC/LC–MS detection and metabolite database analysis showed that flavonoids in Gbtc 2 metabolites were diverse and abundant (Bampali et al., 2021). As a prerequisite for the synthesis of flavonoids, the addition of phenylalanine in the medium can promote the production of flavonoids by Gbtc 2 (Liu et al., 2022; Wang S. et al., 2022). A total of 71 flavonoids were identified, and the properties and structures of each flavonoid had certain uniqueness. The flavonoids identified also have more applications in the industrial field, such as quercetin can be used as a feed additive for the animal husbandry industry. This was one of the few reports of *Aspergillus* fungi to produce flavonoids.

From previous studies on endophytic fungi of *Ginkgo*, few researchers classified the fermentation broth of endophytic fungi into intracellular and extracellular metabolites for analysis. In this study, metabolites of Gbtc 2 mycelium and culture medium were detected and identified separately. LC–MS results showed that various flavonoid metabolites could be directly mapped to the metabolic process, which strongly proved that Gbtc 2 could synthesize flavonoids. In this experiment, the types and concentrations of Gbtc 2 intracellular and extracellular metabolites were different to some extent. Most flavonoid metabolites gathered intracellularly have glycosylation groups, such as astragalin, trifolin, and cynaroside. However, almost all flavonoid monomers gathered extracellularly contain multiple hydroxyl groups. At present, no relevant reports have discussed the distribution rules of different types of flavonoid derivatives intracellularly and extracellularly, which can provide a certain reference for subsequent studies.

Studies have shown that hydroxylation and glycosylation often significantly improve or promote the solubility, storage, and stability of flavonoids (Xiao et al., 2009). Polyhydroxy can reduce the hydrophobicity of flavonoids, thus hindering the interaction in biofilm (Saleh et al., 2020), so that the polyhydroxy flavonoids produced by Gbtc 2 can better enter the culture medium through the cell membrane (Chunjian et al., 2014). Glycosylation of flavonoids will increase their chemical stability and solubility intracellularly, enabling them to enter the active membrane transport system that recognizes glycosylated compounds but does not recognize their aglycones (Xiao et al., 2014). Furthermore, Glycosylation and hydroxylation of isoflavones have been reported to be associated with protein binding and quenching. Hydroxylation increases binding affinity while glycosylation decreases binding affinity (Jinyao and Fenglian, 2008). It was speculated that glycosylation and hydroxylation play a key role in distinguishing functional specificity and suggest that glycosylated flavonoids and hydroxylated flavonoids may have different usages.

### Gbtc 2 has the potential to become a flavonoid producing engineering strain

Flavonoids are the main source of medicine, cosmetics, and food additives. The availability of flavonoids in plants is affected by seasonal or regional variations and is limited by the low abundance of single compounds in complex mixtures (Zhao et al., 2019). Traditional extraction methods and excessive exploitation have led to the exhaustion of plant resources and environmental pollution. It is difficult to entirely rely on chemical synthesis to generate the complex structure of flavonoids, so they were inefficient and cost-effective (Liu et al., 2021). In recent years, reports on the production of paclitaxel by endophytic fungi have shown that endophytic fungi can produce active substances similar to host plants, which provides a new way for the development and production of medicinal components of *Ginkgo* (Stierle et al., 1993; Ajikumar et al., 2010). With the maturity of synthetic biology and multi-omics, engineering cells to produce natural plant products has achieved great success (Ranganathan and Mahalingam, 2019). Due to the transparent metabolic background and strong genetic operability, microbial cells, such as *Escherichia coli*, *Saccharomyces cerevisiae*, and *Corynebacterium glutamicum*, had been widely used as chassis cells to produce natural plant products (Galanie et al., 2015; Bian et al., 2017; Luo et al., 2019). A direct import of a large number of exogenous genetic fragments into chassis cells usually has a low success rate and the possibility of synthesis of flavonoids. If endophytes were screened from the host tissue, they were highly likely to have some functional genes for the synthesis of flavonoids similar to the host during the long-term co-symbiosis (Zou et al., 2019). Meanwhile, endophytes were also possible to have high expression and high activity of plant functional enzymes and the most suitable reaction environment. The specific metabolic pathways of microorganisms have stringent regulatory mechanisms (Wusheng, 2015). Endophytic fungi, which are eukaryotic cells, may be easier to achieve similar expression regulation with plants than endophytic bacteria.

It is reported that *Arabidopsis thaliana* contains GTPase sequences from Cyanobacteria and α-Proteobacteria through endosymbiotic gene transfer (EGT), which is one of the genetic exchanges between plants and endophytes (I N and M Nengah et al., 2014). The results showed that Gbtc 2 possesses several key functional enzyme genes (*4CL* and *CHS*) and a local flavonoid synthesis pathway similar to the *Ginkgo* host. Gbtc 2 has the potential to be developed as an engineering fungus for flavonoid synthesis. Therefore, if the chassis cell itself has some flavonoid metabolic pathways, it is an ideal choice for flavonoid production. Microbial secondary metabolic pathways usually exist in the form of gene clusters on chromosomes, which is helpful for pathway analysis and modification (Kwon et al., 2021). Moreover, the three *DFR*s cloned from *Ginkgo* showed a unique substrate bias by heterologous expression, in which *GbDFR1* and *GbDFR3* showed a tendency to dihydroquercetin substrate, while *GbDFR2* showed a propensity to convert dihydrokaempferol into leucopelargonidin (Cheng et al., 2018). In this study, while analyzing the genome and metabolites of Gbtc 2 from *Ginkgo* root, we also searched for ideal chassis cells that could synthesize flavonoids, providing theoretical basis for the construction of genetically engineered strains producing flavonoids in the future.

## Conclusion

In this study, the flavonoid synthesis pathway of endophytic fungus Gbtc 2 in *Ginkgo* was re-analyzed and reconstructed based on genomic and metabolic analysis, and the secondary flavonoid metabolites were detected by HPLC/LC–MS. The functional enzymes expressed by these genes constitute an almost complete upstream of flavonoid synthesis, which proves that Gbtc 2 has the ability to synthesize basic C6–C3–C6 flavonoid monomers. Gbtc 2 contains key genes or local secondary metabolite synthesis pathways similar to the *Ginkgo* host. Gbtc 2 promoted the production of flavonoids by adding the appropriate amount of phenylalanine. The relative concentrations of intracellular and extracellular flavonoids are pretty different. Most of the flavonoid substances gathered intracellularly possess glycosylated groups, while the components with much higher concentration extracellularly than intracellularly are almost flavonoid monomers with multiple hydroxyls. The reason may be that binding and quenching of proteins are related to glycosylation and hydroxylation, thus fulfilling different biological functions intracellularly and extracellularly. Furthermore, a variety of substances were targeted to the flavonoid synthesis pathway, demonstrating the flavonoid production capacity of Gbtc 2. This study provides a solid basis to the development of genetically engineered fungi to synthesize flavonoids.

## Data availability statement

The datasets presented in this study can be found in online repositories. The names of the repository/repositories and accession number(s) can be found in the article/Supplementary material.

## Author contributions

XW, KZ, XL, and YL conceived and designed the work. KZ, SF, SZ, and XW performed the experiments. XW and KZ wrote and revised the manuscript and contributed equally to this study. ZD revised the manuscript. All authors contributed to the article and approved the submitted version.

## Funding

This work was supported by the following grants: Chinese National Science and Technology Support Program (2013BAC09B00), National Natural Science Foundation of China (302001124 and 31570113), and Graduate Research and Innovation Project (1053320170629) in Central South University, China.

## Conflict of interest

The authors declare that the research was conducted in the absence of any commercial or financial relationships that could be construed as a potential conflict of interest.

## Publisher’s note

All claims expressed in this article are solely those of the authors and do not necessarily represent those of their affiliated organizations, or those of the publisher, the editors and the reviewers. Any product that may be evaluated in this article, or claim that may be made by its manufacturer, is not guaranteed or endorsed by the publisher.

## Supplementary material

The Supplementary material for this article can be found online at: https://www.frontiersin.org/articles/10.3389/fmicb.2022.972294/full#supplementary-material
