# Supplementary material for: The novel distribution of intracellular and extracellular flavonoids produced by Aspergillus sp. Gbtc 2, an endophytic fungus from Ginkgo biloba root
Source: Front Microbiol. 2022 Oct 26;13:972294. doi: 10.3389/fmicb.2022.972294 (PMC9643780; doi:10.3389/fmicb.2022.972294)
Supplement: Supplementary file 1 [file Data_Sheet_1.docx]

# Supplementary Materials

# Figure


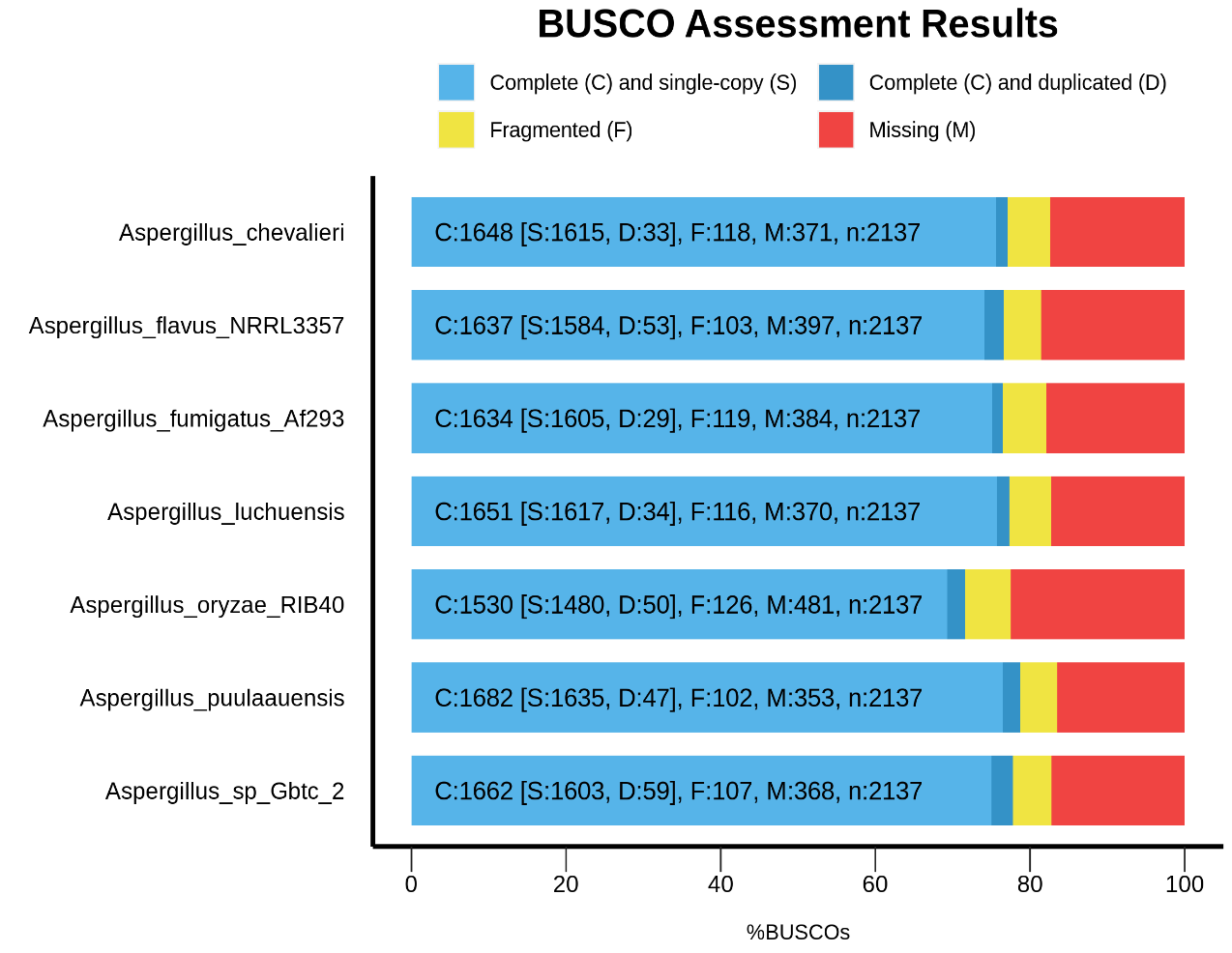


**Figure S1** The BUSCO assessment of *Aspergillus* sp. Gbtc 2 and other six homologous species.


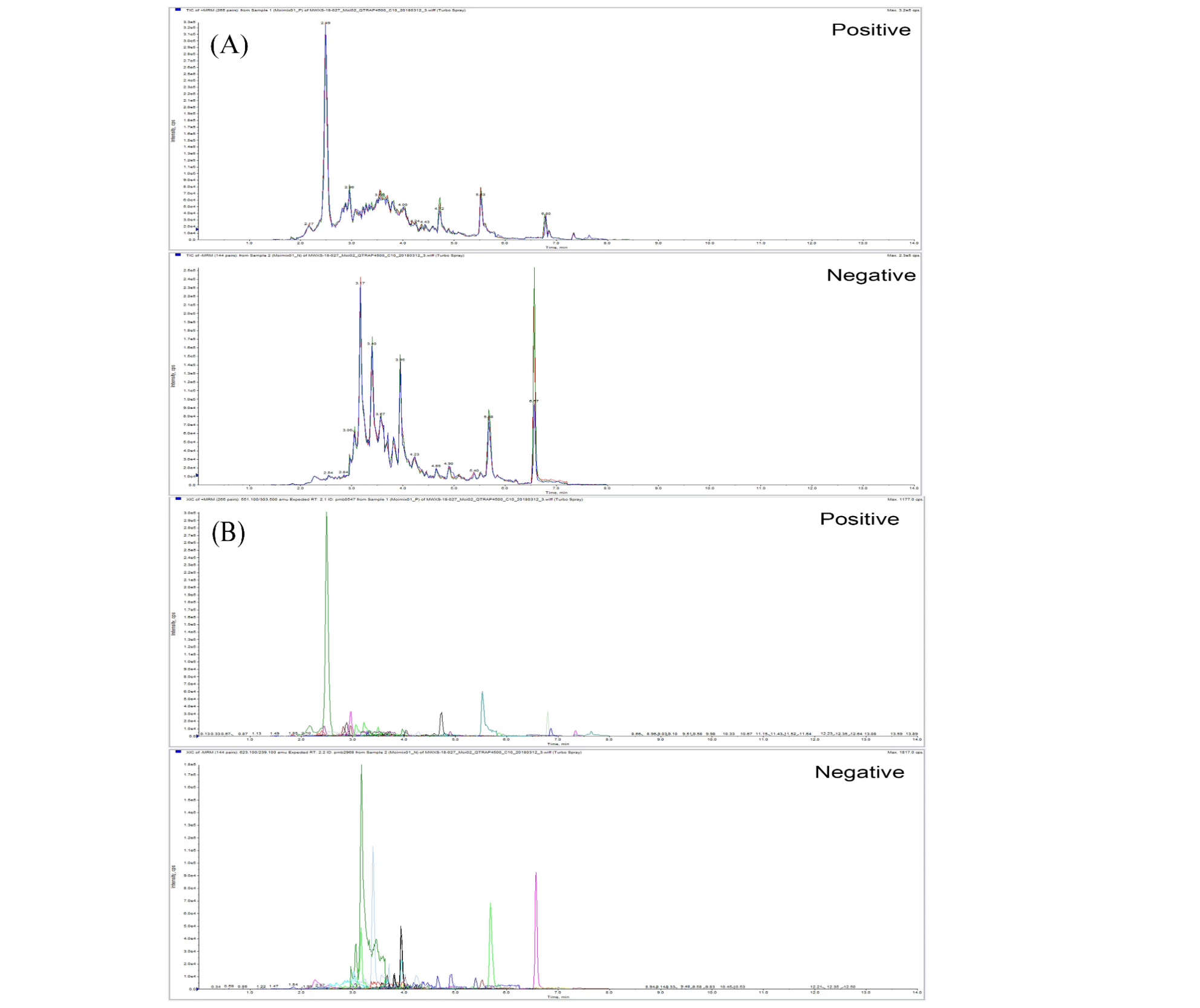


**Figure S****2 (A)** The overlapping analysis of the total ion chromatogram of QC samples. **(B)** The multimodal graph of MRM metabolite detection. The abscissa represents the retention time (RT), and the ordinate represents the intensity of the ion signal.


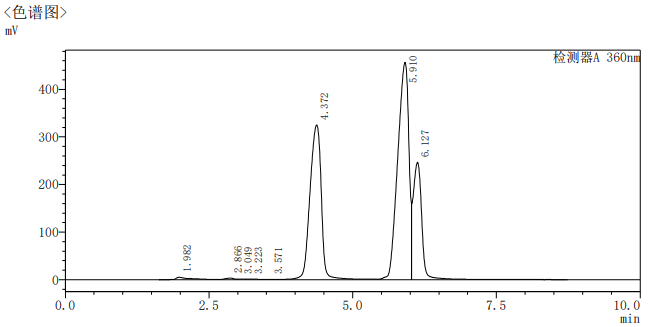


**Figure S3** HPLC results of quercetin, kaempferol and isorhamnetin mixed standards.


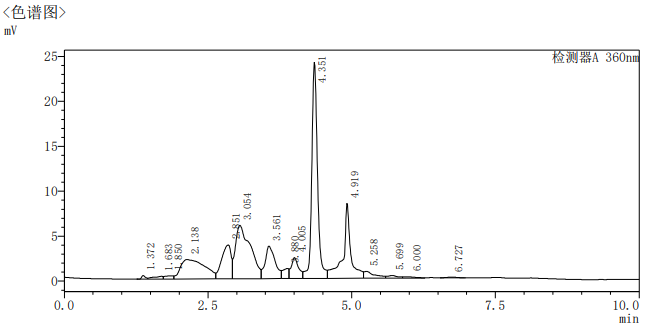


**Figure S4** HPLC results of blank PDB medium.


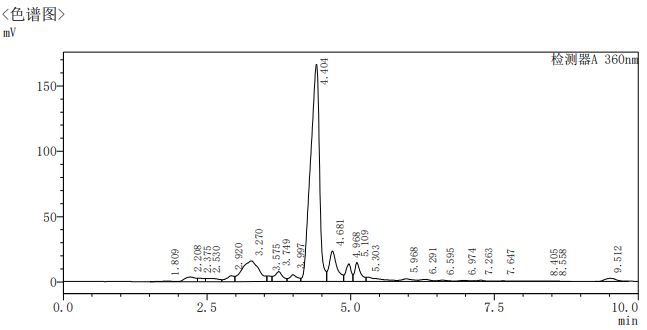


**Figure S5** HPLC results of *Aspergillus* sp. Gbtc2 fermentation broth.

# Table

**Table S1** Sequence information used for phylogenetic tree construction.

| Name | Accession number |
| --- | --- |
| *Aspergillus flavus* NRRL 3357 | XR_005951995.1 |
| *Aspergillus oryzae* JMET 15 | MZ892606.1 |
| *Aspergillus alliaceus* NRRL 4181 | AB002071.1 |
| *Aspergillus puulaauensis* MK2 | AP024448.1 |
| *Penicillium oxalicum* 114-2 | KF152942.1 |
| *Aspergillus pseudoglaucus* WB13 | AB002084.1 |
| *Aspergillus niger* YM33182 | DQ915806.1 |
| *Aspergillus clavatus* NRRL 1 | NG_081374.1 |
| *Aspergillus penicillioides* IFO8155 | AB002078.1 |
| *Aspergillus versicolor* NRRL 238 | NG_067623.1 |
| *Aspergillus ustus* NRRL 275 | AB002072.1 |
| *Aspergillus candidus* MD-3 | EU883597.1 |
| *Neocarpenteles acanthosporus* IFO9490 | AB002075.1 |
| *Aspergillus restrictus* FRR2176 | AB002079.1 |
| *Aspergillus cremeus* NRRL 5081 | NG_063231.1 |
| *Aspergillus glaucus* JCM 1575 | NG_063391.1 |
| *Warcupiella spinulosa* IFO31800 | AB002081.1 |
| *Hamigera ingelheimensis* CBS 163.42 | D14408.1 |
| *Penicillium solitum* 20-01 | JN642222.1 |
| *Monascus purpureus* FRR 1596 | GU733345.1 |
| *Monascus fuliginosus* Q5 | HM188430.1 |
| *Monascus sanguineus* SICC 3.292 | MF372833.1 |
| *Aspergillus terreus* CB10 | OM250078.1 |
| *Aspergillus tubingensis* AtWU_r63 | XR_004775247.1 |
| *Aspergillus wentii* JCM 2724 | AB002063.1 |
| *Aspergillus fumigatus* WL002 | KJ528402.1 |
| *Aspergillus awamori* F2 | MZ359862.1 |
| *Penicillium griseofulvum* 3.5190 | EF608151.1 |
| *Aspergillus flavipes NRRL* 5504 | AB002061.1 |
| *Aspergillus nidulans FGSC* A4 | KY074656.1 |
| *Penicilliopsis clavariiformis* IFO 31626 | AB003946.1 |
| *Penicillium chrysogenum* JMET 24 | MZ892608.1 |
| *Aspergillus sparsus* IAM 13904 | AB002066.1 |

**Table S2** Enzyme genes with gene_id involved in flavonoid metabolism pathway in *Aspergillus* sp. Gbtc 2.

| Enzyme genes | KO number | Gene­_ID |
| --- | --- | --- |
| PAL | K10775 | 5205_g |
| 4CL | K01904 | 1576_g |
| HCT | K13065 | 2182_g; 2702_g; 9404_g |
| CHS | K00660 | 1679_g, 6218_g |
| FLS | K05278 | 9073_g |
| CHI | / | 8262_g |
| F3’M | / | 9416_g |
| LDOX | / | 3683_ g; 7113_ g |
| F3H | / | 1189_g |

**Table S3** The qualitative and relative quantitative table of flavonoids.

| Rt (min) | Mr  (Da) | Ionization  mode | Compounds | Class | Culture medium | Mycelium |
| --- | --- | --- | --- | --- | --- | --- |
| 3.16 | 621.1 | Protonated | Cyanidin O-malonyl-malonylhexoside | F | 2.96E+04 | 8.62E+03 |
| 3.41 | 605.1 | Protonated | Pelargonidin 3-O-malonyl-malonylhexoside | F | 3.00E+04 | 7.56E+03 |
| 4.92 | 302.043 | [M+H]+ | Quercetin | B | 3.18E+04 | 9.00E+00 |
| 3.77 | 594.1585 | [M-H]- | Nicotiflorin | B | 9.00E+00 | 2.98E+04 |
| 2.47 | 772.1 | [M-H]- | Luteolin O-hexosyl-O-hexosyl-O-hexoside | A | 1.33E+03 | 2.83E+04 |
| 3.73 | 610.2 | [M+H]+ | Quercetin 7-O-rutinoside | B | 1.98E+03 | 2.53E+04 |
| 2.81 | 535.1 | Protonated | Cyanidin 3-O-malonylhexoside | F | 1.70E+04 | 1.01E+04 |
| 4.36 | 518 | [M+H]+ | Apigenin O-malonylhexoside | A | 2.57E+04 | 9.00E+00 |
| 4.75 | 254.0579 | [M+H]+ | Daidzein | C | 2.33E+04 | 1.97E+03 |
| 3.82 | 578.1636 | [M-H]- | Isorhoifolin | A | 1.25E+04 | 1.26E+04 |
| 3.45 | 564.1 | [M+H]+ | C-hexosyl-apigenin O-pentoside | E | 1.52E+04 | 9.48E+03 |
| 3.89 | 578.1636 | [M-H]- | Rhoifolin | A | 1.40E+04 | 9.79E+03 |
| 3.32 | 448.1 | [M-H]- | Luteolin C-hexoside | E | 6.37E+02 | 2.23E+04 |
| 4.57 | 254.058 | [M+H]+ | 7,4'-Dihydroxyflavone | A | 2.00E+04 | 2.90E+03 |
| 3.06 | 625.1 | [M-H]- | Luteolin O-hexosyl-O-gluconic acid | A | 2.10E+04 | 1.86E+03 |
| 3.94 | 576.1268 | [M+H]+ | Procyanidin A2 | G | 1.58E+04 | 6.95E+03 |
| 3.68 | 800.1 | [M+H]+ | 6-C-hexosyl-apigenin O-sinapoylhexoside | E | 1.87E+04 | 2.98E+03 |
| 3.6 | 608.1 | [M+H]+ | Chrysoeriol C-hexosyl-O-rhamnoside | E | 1.65E+04 | 5.11E+03 |
| 3.17 | 620.1 | [M-H]- | Cyanidin O-diacetyl-hexoside-O-glyceric acid | F | 1.38E+04 | 7.57E+03 |
| 3.88 | 464.132 | [M-H]- | Hesperetin 5-O-glucoside | D | 6.25E+03 | 1.28E+04 |
| 3.68 | 464.096 | [M-H]- | Isotrifoliin | B | 4.24E+03 | 1.42E+04 |
| 3.45 | 287.24 | Protonated | Cyanidin | F | 1.42E+04 | 3.64E+03 |
| 3.88 | 432.113 | [M+H]+ | Apigenin C-glucoside | E | 1.60E+03 | 1.46E+04 |
| 4.29 | 432.106 | [M+H]+ | Kaempferin | B | 9.00E+00 | 1.61E+04 |
| 2.75 | 786.1 | [M+H]+ | Chrysoeriol 6-C-hexoside 8-C-hexoside-O-hexoside | E | 9.51E+03 | 5.27E+03 |
| 3.91 | 638.1 | [M+H]+ | Tricin 5-O-rutinoside | A | 1.29E+04 | 1.39E+03 |
| 2.93 | 756.1 | [M+H]+ | Luteolin C-hexosyl-O-rhamnoside O-hexoside | E | 5.48E+03 | 8.30E+03 |
| 3.38 | 580.1 | [M+H]+ | 8-C-hexosyl-luteolin O-pentoside | E | 1.24E+04 | 1.27E+03 |
| 2.15 | 697.1 | Protonated | Cyanidin 3-O-glucosyl-malonylglucoside | F | 8.95E+03 | 4.31E+03 |
| 4.04 | 464.096 | [M+H]+ | Spiraeoside | B | 9.00E+00 | 1.25E+04 |
| 3.66 | 288.063 | [M+H]+ | Fustin | B | 9.04E+03 | 3.26E+03 |
| 2.84 | 624.2 | [M+H]+ | 6-C-hexosyl chrysoeriol O-hexoside | E | 7.83E+03 | 4.19E+03 |
| 2.77 | 640.1 | [M+H]+ | C-hexosyl-isorhamnetin O-hexoside | E | 5.43E+03 | 6.53E+03 |
| 3.67 | 576.1268 | [M+H]+ | Procyanidin A1 | G | 8.02E+03 | 3.78E+03 |
| 5.3 | 272.069 | [M-H]- | Butein | D | 8.88E+03 | 2.36E+03 |
| 2.8 | 610.2 | [M+H]+ | C-hexosyl-luteolin O-hexoside | E | 8.39E+03 | 2.73E+03 |
| 6.19 | 316.058 | [M-H]- | Rhamnetin | B | 1.03E+04 | 9.00E+00 |
| 4.97 | 302.043 | [M-H]- | Morin | B | 2.09E+03 | 7.63E+03 |
| 2.41 | 466.1 | [M-H]- | Cyanidin O-syringic acid | F | 6.40E+03 | 3.01E+03 |
| 3.94 | 304.058 | [M-H]- | Taxifolin | B | 7.16E+03 | 9.00E+00 |
| 4.38 | 724.2 | [M+H]+ | Tricin O-rhamnosyl-O-malonylhexoside | A | 4.47E+03 | 2.30E+03 |

A. Flavone; B. Flavonol; C. Isoflavone; D. Flavanones; E. Flavone C-glycosides; F. Anthocyanins; G. Proanthocyanidins

**Table S4** Effect of phenylalanine addition on flavonoids content produced by *Aspergillus* sp. Gbtc 2 and blank control group.

| Phenylalanine addition (g/L) | Q (μg/mL) | E(yEr±) | K (μg/mL) | E(yEr±) | C (μg/mL) | E(yEr±) |
| --- | --- | --- | --- | --- | --- | --- |
| CK | 16.2672 | 1.4090 | 0.0121 | 0.0210 | 40.8627 | 3.5028 |
| 0 | 68.1733 | 1.6033 | 10.6600 | 1.1262 | 199.2575 | 2.8932 |
| 1 | 141.0800 | 1.7056 | 13.1533 | 0.8201 | 388.8356 | 5.5843 |
| 2 | 176.6467 | 1.9731 | 15.4267 | 0.4868 | 484.1095 | 4.1879 |
| 5 | 69.6333 | 0.6886 | 10.0400 | 1.1433 | 201.2853 | 3.5401 |
| 10 | 53.1467 | 2.5726 | 7.4800 | 0.7373 | 153.1453 | 6.8634 |
